# Robotic surgery in Cardiology: a safe and effective procedure

**DOI:** 10.1590/S1679-45082013000300007

**Published:** 2013

**Authors:** Robinson Poffo, Alisson Parrilha Toschi, Renato Bastos Pope, Alex Luiz Celullare, Anderson Benício, Claudio Henrique Fischer, Marcelo Luiz Campos Vieira, Alexandre Teruya, Dina Mie Hatanaka, Gabriel Franzin Rusca, Marcia Makdisse

**Affiliations:** 1 Hospital Israelita Albert Einstein, São Paulo, SP, Brazil

**Keywords:** Cardiac surgery, Robotics, Myocardial revascularization, Mitral valve, Pericardium, Heart septal defects, atrial, Heart defects, congenital, Surgical procedures, minimally invasive, Atrial fibrillation

## Abstract

**Objective:**

To evaluate the short and medium-term outcomes of patients undergoing robotic-assisted minimally invasive cardiac surgery.

**Methods:**

From March 2010 to March 2013, 21 patients underwent robotic-assisted cardiac surgery. The procedures performed were: mitral valve repair, mitral valve replacement, surgical correction of atrial fibrillation, surgical correction of atrial septal defect, intracardiac tumor resection, totally endoscopic coronary artery bypass surgery and pericardiectomy.

**Results:**

The mean age was 48.39±18.05 years. The mean cardiopulmonary bypass time was 151.7±99.97 minutes, and the mean aortic cross-clamp time was 109.94±81.34 minutes. The mean duration of intubation was 7.52±15.2 hours, and 16 (76.2%) patients were extubated in the operating room immediately after the procedure. The mean length of intensive care unit stay was 1.67±1.46 days. There were no conversions to sternotomy. There was no in-hospital death or deaths during the medium-term follow-up. Patients mean follow up time was 684±346 days, ranging from 28 to 1096 days.

**Conclusion:**

Robotic-assisted cardiac surgery proved to be feasible, safe and effective and can be applied in the correction of various intra and extracardiac pathologies.

## RESUMO

**Objetivo:**

Avaliar os resultados em curto e médio prazo dos pacientes submetidos à cirurgia cardíaca minimamente invasiva robô-assistida.

**Métodos:**

De março de 2010 a março de 2013, 21 pacientes foram submetidos à cirurgia cardíaca robô-assistida. Os procedimentos realizados foram: plastia valvar mitral, troca valvar mitral, cirurgia de correção da fibrilação atrial, correção cirúrgica da comunicação interatrial, ressecção de tumor intracardíaco, revascularização do miocárdio totalmente endoscópica e pericardiectomia.

**Resultados:**

A idade média foi de 48,39±18,05 anos. 0 tempo médio de circulação extracorpórea foi de 151,7±99,97 minutos, e o tempo médio de pinçamento aórtico foi de 109,94±81,34 minutos. 0 tempo médio de intubação orotraqueal foi de 7,52±15,2 horas, sendo que 16 (76,2%) pacientes foram extubados ainda em sala operatória, imediatamente após o procedimento. 0 tempo médio de permanência em unidade de terapia intensiva foi de 1,67±1,46 dias. Não houve conversões para esternotomia. Não houve óbito intra-hospitalar ou mesmo durante o seguimento em médio prazo dos pacientes. A média do tempo de acompanhamento dos pacientes foi de 684±346 dias, variando de 28 dias a 1096 dias.

**Conclusão:**

A cirurgia cardíaca robô-assistida mostrou-se exequível, segura e efetiva, podendo ser aplicada na correção de diversas patologias intra e extracardíacas.

## INTRODUCTION

Robotic-assisted totally endoscopic cardiac surgery has been performed for more than a decade in Europe and in the United States of America^(1–3)^. Since the first procedures, much has been discussed about its feasibility, safety and, moreover, its results^(4)^. A growing involvement of large centers with this new modality has been observed throughout these years of development. One of the major reasons of motivation is the reduced surgical trauma, because a smaller amount of tissue is sectioned and/or manipulated. Other characteristics of the technique are the shorter length of hospital stay associated with the perception of greater patient satisfaction and earlier return to their social and professional activities in comparison to the conventional open procedures^(4)^.

## OBJECTIVE

To evaluate the short and medium-term outcomes of patients undergoing robotic-assisted cardiac surgery in a general, tertiary-care, philanthropic hospital.

## METHODS

All participants gave written informed consent. They were given information on the surgical possibilities regarding the performance of the procedure and chose the robotic-assisted approach. The study was approved by the *Hospital Israelita Albert Einstein* ethics committee under number CEP/Einstein 11/1501, entitled “Einstein Registry of Robotic-assisted Minimally Invasive Cardiovascular Surgery”. Data were prospectively collected and recorded in a data basis exclusively designated for this project.

The procedures were performed from March 2010 to March 2013. Inclusion criteria followed the usual indications for conventional surgical correction of cardiac pathologies whether acquired or congenital. Exclusion criteria for the performance of the robotic-assisted procedures were: thoracic deformities; severe thoracic trauma; anatomical or pathological abnormalities of the peripheral vascular system; moderate or severe aortic regurgitation; ejection fraction <0.40%; and, for totally endoscopic coronary artery bypass (TECAB), coronary arteries with a diameter <1.5mm or calcified were excluded.

Twenty one patients underwent robotic-assisted cardiac surgery. The procedures performed were: mitral valve repair, mitral valve replacement, surgical correction of atrial fibrillation, surgical correction of atrial septal defect, intracardiac tumor resection, TECAB, and pericardiectomy.

All patients were operated on using the DaVinci™ system (Intuitive Surgical, Inc., Sunnyvale, CA, USA), composed of a set of four robotic arms, an image capture and recording system, and a console through which the surgeon controls the robot movements.

### Surgical technique

#### Mitral valve operations

For robotic mitral valve repair or replacement, the technique used was previously described by Chitwood et al.^(5)^. Patients were intubated using a Robert Shaw endotracheal tube for selective pulmonary ventilation and positioned with the right hemo-thorax elevated at 20°. Disposable paddles for external cardiac defibrillation were placed in the right scapular and anterolateral regions of the left hemi-thorax. A nasopharyngeal thermometer for body temperature control and a three-dimensional transducer for intraoperative transesophageal echocardiography were used.

**Figure 1:**
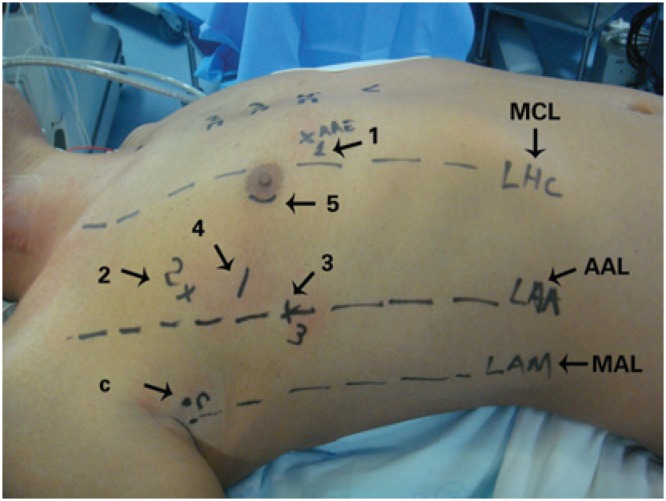
Preoperative markings and sites for the introduction of (1) left atrial retractor; (2) left robotic arm; (3) right robotic arm, (4) working trocar; (5) micro camera. C: transthoracic aortic clamp; MCL: mid-clavicular line; AAL: anterior axillary line; MAL: mid-axillary line

**Figure 2:**
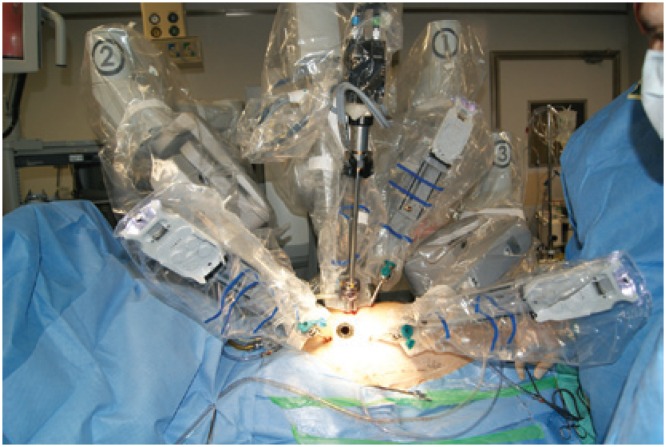
External aspect of the operative field: DaVinci™ robotic system docked to the patient

After positioning the patient, markings were made on the thorax surface for trocar introduction (Figure 1). After systemic heparinization, peripheral cannulation of the femoral vein and artery as well of the right internal jugular vein were performed oriented by transesophageal echocardiography. Prior to trocar introduction, the right lung was selectivated. The trocar for the micro camera was positionated and the micro camera was introduced. The right hemi-thorax was inspected and then the remaining trocars were introduced. The DaVinci™ robotic system (Intuitive Surgical, Inc., Sunnyvale, CA, USA) was docked (Figure 2).

Through the second right intercostal space (RICS) in the mid-axillary line (MAL), the Chitwood transthoracic clamp was introduced (Fehling Instruments GMBH & CO. KG, Karlstein, Germany). Extracorporeal circulation (ECC) was established and the patient was cooled down to 28°C. With the aid of videothoracoscopy, the ascending aorta was clamped and punctured with a 30-cm metal needle (Geister Medizintechnik, Tuttlingen, Germany) for the administration of antegrade crystalloid cold cardioplegic solution Custodiol HTK (Köhler Chemie GmbH, Bensheim, Germany). From left atrial opening to closure, CO_2_ was insufflated with the purpose of reducing the possibility of air embolism.

The mitral valve was inspected prior to the procedure and then repaired or replaced. Under transesophageal echocardiographic monitoring, the heart was properly de-aired, and ECC was discontinued. The femoral and jugular cannulas were removed, and heparin was reverted using protamine at a 1:1 ratio. The right hemi-thorax was drained and the drain was exteriorized through the trocar port of the right robotic arm, directed to the interior of the pericardial sac.

One of the patients had atrial fibrillation associated with the mitral valve disease, and underwent mitral valve plasty associated to epicardial ablation with electrical isolation of the pulmonary veins using the Cobra Adhere XL (Estech, San Ramon, CA, USA) system, as described by Bevilaqua et al.^(6)^, prior to the establishment of ECC.

#### Correction of atrial septal defect

The robotic technique described by Poffo et al.^(7)^ was used in the cases of surgical correction of atrial septal defect (ASD). The approach is very similar to that used in the robotic-assisted mitral valve repair^(8)^. In the case of ASD correction, the chamber approached is the right atrium, after isolation of the superior and inferior venae cavae. With the introduction of the micro camera in the right atrium, ASD was visualized and then corrected using a bovine pericardial patch. In the cases of this serie, all ASDs were ostium secundum type.

#### Intracardiac tumor resection

In the cases of robotic-assisted resection of intracardiac tumors, the surgical approach was similar to those described in the robotic-assisted surgery of the mitral valve and correction of ASD^(9)^. In the cases in which the tumor were located in the left atrium, left atriotomy was performed in the region of the right pulmonary veins following cardioplegia. In one of the cases, the tumor was attached to the left posterior atrial wall, which was broadly resected and replaced by a bovine pericardial patch. In another case, the tumor was adhered to the atrioventricular septum, anterior to the anterior cusp of the mitral valve. In the case in which the tumor was located in the right atrium, this chamber was opened and, without using cardioplegia, part of the right atrial free wall in which the tumor was adhered to was resected, and primary suture of the right atrium was performed.

#### Pericardiectomy

For robotic-assisted pericardial resection, the technique used was similar to the one used for mitral valve repair, and the procedure was performed off-pump. The pericardium was broadly resected, parallel to the right phrenic nerve up to the region corresponding to the anterior aspect of the left ventricle.

#### Treatment of atrial fibrillation

Patient preparation, positioning and markings are the same as described for mitral valve surgery.

Prior to the introduction of the trocars, the right lung was isolated. The trocar for the micro camera was positionated and the micro camera was introduced in the fourth RICS, in the anterior axillary line (AAL). The right thorax was inspected and then the remaining trocars were introduced. The DaVinci™ robotic system (Intuitive Surgical, Inc., Sunnyvale, CA, USA) was docked.

After opening the pericardial sac, the superior and inferior venae cavae were dissected and a guide was introduced through the transverse sinus, posterior to the left atrium, right below the pulmonary veins, until emerging in the oblique sinus. The ablation probe Cobra Adhere XL (Estech, San Ramon, CA, USA) was then introduced using a guide, and it was positioned for ablation. After correctly positioned via endoscopic approach, the epicardial ablation system was activated. After the first ablation, the ablation line was visually checked and, later, electrically checked using the electroanatomical system. If a gap was identified by the electroanatomical system it was promptly corrected by the eletrophisiologist.

#### Totally endoscopic coronary artery bypass

**Figure 3:**
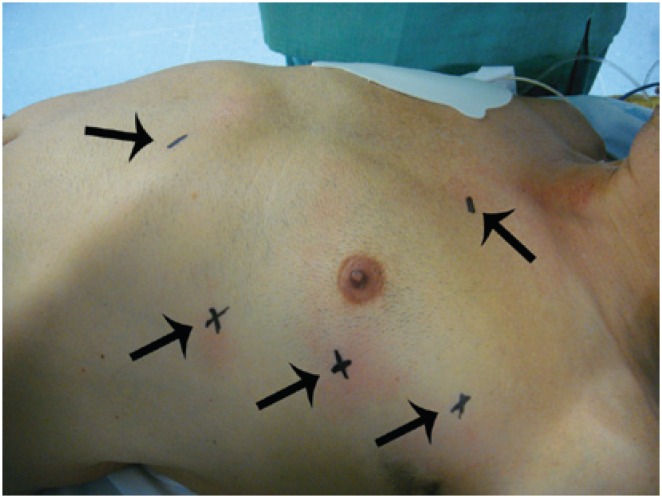
Arrows indicate the markings for the introduction of trocars for totally endoscopic coronary artery bypass

The technique described by Bonatti^(10)^ was used for totally endoscopic coronary artery bypass (TECAB) (Figure 3). TECAB was performed off-pump via left thoracic approach.

After single-lung ventilation had been established with isolation of the left lung, a 12-mm trocar was introduced in the fifth left ICS (LICS), for the introduction of the micro camera. Two trocars for the 8-mm robotic instruments were inserted in the third and seventh LICS in the left AAL. CO_2_ was constantly insufflated in the left thorax thus ensuring room for dissection of the left internal thoracic artery (LITA).

After robotic dissection of the LITA a robotic coronary stabilizer was used, which was introduced in the left hemithorax and connected to the fourth robotic arm. The coronary artery segment to be anastomosed was mechanically immobilized and occluded proximally and distally using a specific tourniquet. After completion of the anastomosis, a TTFM (Medstim, Oslo, Norway) flowmeter was used to check proper flow through the anastomosis.

### Statistical analysis

Continuous variables were expressed as means and standard deviation. The Kaplan-Meier method was used for survival analysis. The GraphPad Prism 6.02 software program (GraphPad Software Inc, San Diego, CA, USA) was also used.

## RESULTS

During the study period, 21 patients were operated on. Thirteen (72.2%) patients were males. The mean age was 48.39±18.05 years, ranging from 22 to 81 years. The procedures were successfully performed in all patients (Table 1). Of the 21 patients, 9 (42.8%) underwent mitral valve surgery (7 valvuloplasty and 2 valve replacement); in 2 (9.5%) cases, patent foramen ovale was found, and atrial septal suture was performed. In one case, mitral valve repair was associated with epicardial ablation for the correction of atrial fibrillation. After the procedures, all patients underwent intraoperative transesophageal echocardiography, which showed successful valvuloplasty; in the cases of valve replacement, the prostheses were well positioned and functioning normally. Three (14.3%) patients underwent intracardiac tumor resection (one right atrial tumor and two left atrial tumors). Four (19%) underwent ASD correction using the atrial septoplasty technique with bovine pericardial patch. Three (14.3%) patients underwent totally endoscopic coronary artery bypass. Of these, two patients had already undergone previous angioplasty and progressed with intracoronary stent stenosis; one patient had a severe lesion in the proximal third of the left anterior descending artery. The procedures were using beating heart and off pump techniques. LITA grafts used to revascularize the left anterior descending artery in the three patients. After completion of the anastomosis, a TTFM flowmeter (Medstim, Oslo, Norway) was used to check proper blood flow through the anastomosis.

**Table 1:** Procedures performed

Patient	Gender	Age (years)	Diagnosis	Treatment
1	F	34	ASD	Atrial septoplasty
2	M	39	ASD	Atrial septoplasty
3	M	53	MiR + PFO	Mitral valve replacement + PFO closure
4	M	51	MiR	Mitral valve repair + annuloplasty
5	F	38	MiR + PFO	Mitral valve replacement + PFO closure
6	F	22	LA tumor	LA tumor resection
7	M	54	MiR	Mitral valve repair + annuloplasty
8	M	67	MiR	Mitral valve repair + annuloplasty
9	M	42	MiR	Mitral valve repair + annuloplasty
10	F	60	MiR	Mitral valve repair + annuloplasty
11	F	24	ASD	Atrial septoplasty
12	M	77	MiR + AF	Mitral valve repair + annuloplasty + epicardial ablation
13	M	51	AF	Hybrid AF treatment
14	F	25	RA tumor + SVC thrombus	RA tumor resection + SVC thrombectomy
15	M	49	Coi	Coronary artery bypass (LITA - LAD)
16	M	72	ICo	Coronary artery bypass (LITA - LAD)
17	M	32	MiR	Mitral valve repair + annuloplasty
18	M	81	Constrictive pericarditis	Pericardiectomy
19	M	57	Coi	Coronary artery bypass (LITA - LAD)
20	F	44	ASD	Atrial septoplasty
21	F	40	LA tumor	LA tumor resection

F: female; ASD: atrial septal defect; M: male; MiR: mitral regurgitation; PFO: patent forame ovale; LA: left atrium; AF: atrial fibrillation; RA: right atrium; SVC: superior vena cava; Coi: coronary insufficiency; LITA: left internal thoracic artery; LAD: left anterior descending artery.

One (4.8%) patient underwent hybrid treatment of atrial fibrillation epicardial-isolation of the pulmonary veins associated with endocardial ablation using electroanatomical mapping with a CARTO 3D™ (Biosense Webster, Johnson & Johnson, Diamond Bar, California, USA) device, and another (4.8%) patient underwent pericardiectomy.

The mean cardiopulmonary bypass time was 151.7±99.97 minutes and the mean aortic cross-clamp time was 109.94±81.34 minutes. The mean tracheal intubation time was 7.52±15.2 hours, and 16 patients were extubated in the operating room immediately after the procedure. The mean intensive care unit (ICU) length of stay was 1.67±1.46 days (Table 2).

**Table 2:** Data on the hospital course

Course		
Aortic cross-clamp time, minutes	109.94±81.34	(0-260)
ECC time, minutes	151.7±99.97	(0-340)
TI time, hours	7.52±15.2	(0- 48)
Days in ICU	1.67±1.46	(1-6)
Length of hospital stay, days	5.59±3.78	(3-15)

ICU: intensive care unit; ECC: extracorporeal circulation; TI: tracheal intubation.

The major complications were: unilateral pulmonary edema (UPE, 1 patient), atrial fibrillation with rapid ventricular response (1 patient) and systemic inflammatory response syndrome (SIRS, 1 patient).

All procedures were performed as planned, with no need for conversion to sternotomy. No problems regarding the use of the robot or peripheral cannulation occurred. No case of stroke was observed.

**Figure 4:**
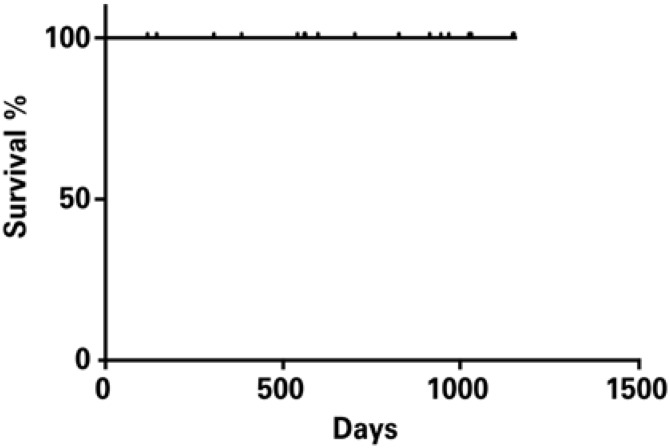
Kaplan-Meier survival curve of operated patients

All patients operated on using the robot-assisted minimally invasive technique are alive (Figure 4) and being followed up on an outpatient basis. Patients mean follow up time was 684±346 days, ranging from 28 to 1096 days.

## DISCUSSION

The use of minimally invasive cardiac surgery via minithoracotomy was, for many years, limited by the need for direct visualization of the heart and by the use of conventional surgical instruments used in complete sternotomy. With the incorporation of the video-assisted techniques, minimally invasive surgery proved to be safe and effective in different surgical situations^(11–14)^. However, learning is required for the acquisition of new abilities in the manipulation of long instruments in a restricted operative field in which direct vision is frequently replaced by a two dimensional vision that is seen in the video monitor and by the change in the tactile feedback provided by the direct contact with tissues. One of the major technological advances in the surgical area was the introduction of the DaVinci™ robotic system, which permits significant improvement of what can be visualized in the operative field, by means of three-dimensional image capture, magnified by ten times. This adds more precision to the procedure and allows cardiac surgeries to be performed using even smaller incisions.

The robot-assisted minimally invasive surgery, with a high-definition three-dimensional visual field and very sophisticated micro-articulated instruments have markedly influenced the setting of minimally invasive surgery. Several studies have demonstrated its efficacy and safety in different medical specialties^(15)^.

Broader movements of the articulated instruments in different angles and directions not only permit refined movements, but allows working in a limited space such as the atrial cavity.

Since the first robotic-assisted mitral valve repair was performed, back in 1998, independently by Carpentier in France and by Mohr in Germany, this type of procedure has been gaining popularity^(14)^ and is the most frequently performed robotic-assisted cardiac surgery today.

Robotic-assisted mitral valve surgery currently offers complete anatomical correction of all categories of prolapse. The idea that robotic-assisted mitral repair limits the ability to perform a complete anatomical correction or that it can be associated with a higher risk of severe adverse events is increasingly contrary to our findings and, moreover, to current results from the literature^(16)^.

In this case series, the robotic technique was also used for the correction of ASD in four cases, which had a short length of hospital stay and no complications. In addition to the functional gain with an earlier return to daily activities because sternotomy was not performed, there is a secondary aesthetic gain, especially in women. Today, this is our preferred approach in the correction of this congenital heart disease.

In three cases of intracardiac tumors, the robotic-assisted technique permitted complete resection of these masses without the need for sternotomy. One of the patients was obese, with a body mass index of 32kg/m^2^. We observed that obesity was not a contraindication for the procedure; on the contrary, the procedure has the great advantage of allowing early mobilization of the patient, who can get out of bed and walk within the first 24 hours.

Regarding robotic-assisted pericardiectomy, the technique can be easily used since, from the surgical point of view, it represents one of the first steps in other robotic-assisted cardiac procedures. The major limitation is believed to lie in significantly calcified pericardia since the robotic instruments are extremely delicate, thus sectioning these tissues would probably be difficult.

One patient underwent hybrid ablation of atrial fibrillation (AF). This procedure was chosen because the patient had persistent AF and had already undergone two endocardial ablation procedures via cardiac catheterization unsuccessfully. He had been followed up for 18 months and has a normal sinus rhythm and is not taking any antiarrhythmic medication or oral anticoagulants. From the technical point of view, the robotic equipment greatly facilitates the exposure of the venae cavae and left atrium, which is fundamental to perform the epicardial ablation.

In the three cases of totally endoscopic coronary artery bypass grafting, the left internal thoracic artery was used to revascularize the left anterior descending artery. The results obtained were comparable to those of the literature^(17)^. The patients had uneventful postoperative courses and all were discharged on the second postoperative day.

As for conventional coronary artery bypass, patient selection is fundamental for the success of TECAB. In addition, the strategy of choosing the grafts should never compromise complete revascularization. Other considerations are also relevant: patients with pulmonary disease or ventricular dysfunction should be judiciously selected because CO_2_ insufflation ultimately decreases venous return because of the increase in the intrathoracic pressure, and thus can worsen the hemodynamic performance; the location, quality and course of the target artery are more important in TECAB than in conventional surgery because it may be difficult to find it sometimes, especially if it is located inside the epicardial fat or even if it is an intramyocardial artery.

At first, TECAB seems to be extremely selective, although very useful. For instance, in the present case series, two patients had already underwent percutaneous coronary angioplasty whit a stent of the left anterior descending artery and one of them had had intrastent stenosis of the left anterior descending artery twice. All the coronary bypasses had been made with LITA grafts and were uneventful.

However, TECAB still has some limitations, especially in multivessel disease cases. Approaching the aorta for venous grafts is still a problem. For this, the great advance in TECAB is the use of multiple arterial grafts, especially those composed in “artificial y” and sequential anastomoses^(17)^. Another important aspect in the evolution of TECAB is the development of hybrid procedures, in multivessel disease cases.

Regarding the complications found in the immediate postoperative period, UPE occurred in one patient undergoing mitral valve repair. The etiology of this edema as a result of unipulmonary ventilation is speculative^(18)^. It is believed to result from increased pulmonary capillary permeability. In this case, treatment consisted of ventilatory support for 24 hours. This patient was discharged in the 6th postoperative day. In the SIRS case (mitral valve replacement), the patient had significant hemorrhage due to a coagulation disorder and progressed to distributive shock. He was treated with vasoactive drugs and ventilatory support with protective strategy for 24 hours. His length of hospital stay was the longest in this case series (15 days). One patient (intracardiac tumor ressection) had atrial fibrillation with rapid ventricular response, which was chemically reverted with intravenous amiodarone at a loading dose; oral maintenance was given for one month. Studies in the literature show that the rate of this complication in the postoperative period of cardiac surgery depends on the pathology operated, ranging from 5% to 40% in coronary artery bypass grafting cases, and from 37% to 50% in patients undergoing heart valve surgery^(19)^. In this cohort of 21 patients, only one had postoperative paroxysmal AF (4.7%).

The relatively longer operative time of robotic-assisted procedures is not related to increased morbidity or complications. In this case series, a short length of hospital stay was observed (5.59±3.78 days), in addition to a very short intubation time (7.52±15.2 hours) and length of intensive care unit stay (1.67±1.46 days).

This study showed that the method is feasible and safe, with good surgical outcomes associated with excellent secondary aesthetical results.

## CONCLUSION

After analyzing these results, we can conclude that robotic-assisted minimally invasive cardiac surgery is feasible, safe and effective, and can be applied in the correction of several intra and extracardiac pathologies.
